# The Role of Bovine and Non-Bovine Milk in Cardiometabolic Health: Should We Raise the “Baa”?

**DOI:** 10.3390/nu14020290

**Published:** 2022-01-11

**Authors:** Jack Penhaligan, Sally D. Poppitt, Jennifer L. Miles-Chan

**Affiliations:** 1 Human Nutrition Unit, School of Biological Sciences, University of Auckland, Auckland 1024, New Zealand; jpen110@aucklanduni.ac.nz (J.P.); s.poppitt@auckland.ac.nz (S.D.P.); 2 High Value Nutrition, National Science Challenge, Auckland 1023, New Zealand; 3 Riddet Centre of Research Excellence (CoRE) for Food and Nutrition, Palmerston North 4474, New Zealand

**Keywords:** milk, cardiometabolic health, metabolism, glycaemia, energy expenditure, appetite, obesity, type II diabetes

## Abstract

Although causality is yet to be confirmed, a considerable volume of research has explored the relationships between cow milk consumption, type II diabetes, and cardiovascular disease. Contrastingly, it has not been comprehensively examined whether milk of non-bovine origin can provide cardiometabolic protection. This narrative review outlines the marked differences in macronutrient composition, particularly protein and lipid content, and discusses how whole milk product (and individual milk ingredients) from different species could impact cardiometabolic health. There is some data, although primarily from compositional analyses, animal studies, and acute clinical trials, that non-bovine milk (notably sheep and goat milk) could be a viable substitute to cow milk for the maintenance, or enhancement, of cardiometabolic health. With a high content of medium-chain triglycerides, conjugated linoleic acid, leucine, and essential minerals, sheep milk could assist in the prevention of metabolic-related disorders. Similarly, albeit with a lower content of such functional compounds relative to sheep milk, goat and buffalo milk could be plausible counterparts to cow milk. However, the evidence required to generate nutritional recommendations for ‘non-bovine milk’ is currently lacking. Longer-term randomised controlled trials must assess how the bioactive ingredients of different species’ milks collectively influence biomarkers of, and subsequently incidence of, cardiometabolic health.

## 1. Cow Milk Consumption and Cardiometabolic Health

The consumption of cow dairy products is a dominant feature in the diet of many cultures globally, particularly among Western communities. There is some evidence from epidemiological studies and systematic reviews alike that dairy intake is inversely linked with the risk of developing metabolic syndrome [1,2,3]. More pertinently, a body of data supports a negative association between milk intake and the risk of developing dysglycaemia, dyslipidaemia, and hypertension [1,4]. However, with gold-standard data from long-term randomised controlled trials (RCTs) featuring type II diabetes (T2D) and cardiovascular disease (CVD) incidence as primary endpoints not currently available, the causality of these findings remains to be confirmed [5]. Nonetheless, putative explanations for a possible metabolic syndrome risk reduction include a direct modulation of the glycaemic response [2,6], and an indirect modulation of body weight through upregulation of postprandial thermogenesis [6,7,8] and/or suppression of appetite [9,10,11]. Features of, or responses to, milk that might contribute to any cardiometabolic protection include the bioactive peptide content [12,13]; fatty acid (FA) content [14], e.g., conjugated linoleic acid (CLA) [15]; glycaemic index (GI) [16,17]; promotion of satiety [18]; mineral content, particularly calcium, magnesium, and potassium [19,20,21,22]; and folate bioavailability [23].

Although there is growing data on the acute and chronic health benefits of cow milk, albeit not yet conclusive, whether milk from alternative (non-bovine) sources could provide comparable or superior cardiometabolic protection has not yet been comprehensively reviewed.

## 2. Cow Milk Alternatives

The worldwide commercial production of cow milk decisively eclipses the relatively minor contributions from alternative animal species (Table 1). Nonetheless, these milks remain valuable primary sources of nutrition for many countries and communities globally.

Owing to the specific make-up of proteins (e.g., β-lactoglobulin; β-lg) and sugars (e.g., lactose) within cow milk, the global prevalence of cow milk allergy and intolerance is notably high. Approximately 65% of adults worldwide have a suboptimal capacity to digest and absorb lactose [25]. In Asian and American Indian populations, the reported prevalence of lactose intolerance is closer to 100% [26,27]. However, with marked compositional differences, hypoallergenicity and improved tolerability have been indicated following the ingestion of goat [28], sheep [29], camel [30], buffalo [31], and donkey [32] milk, as compared to cow milk. It should be noted that throughout this review buffalo milk refers to the produce of animals of the *Bubalus* genus.

Lastly, non-dairy substitutes for milk, including soy, oat, rice, and nut ‘milk beverages’ have received growing attention. These plant-based alternatives are formulated through the disintegration of plant material, extraction in water, and subsequent homogenisation, which produces a ‘milk’ reminiscent of the consistency and appearance of animal milk [33]. Despite a typically substandard macronutrient profile relative to mammalian milk, plant-based ‘milks’ possess distinct functional ingredients, lower allergenicity and greater affordability, which have impelled a noticeable surge in demand and production.

## 3. The Composition and Digestibility of Milks of Different Origin

### 3.1. Composition

**Figure 1:**
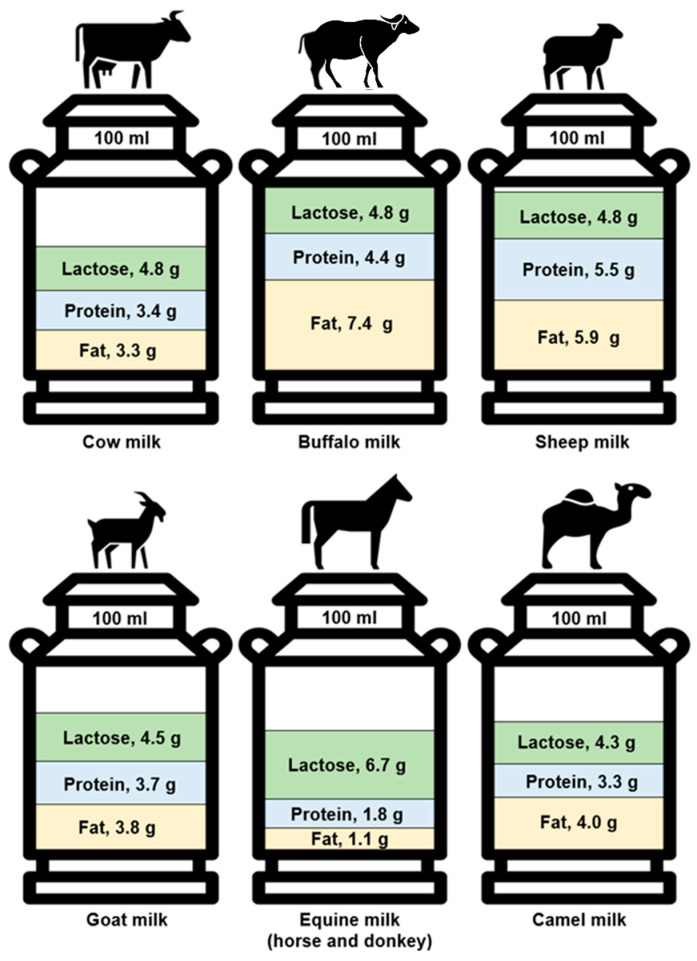
The composition of different species’ milk by fat, protein, and lactose content per 100 mL [22,29,34,35]. Equine milk values represent the mean nutrient content in mare and donkey milks.

With knowledge of varying nutritional profiles, beyond any implications of a reduced allergenic potential of non-bovine milk, the ingestion of milk from different species could also engender distinct health benefits. Marked differences have been documented in macronutrient composition between multiple milk sources, particularly in terms of protein and lipid content. For instance, 100 g of sheep milk provides a markedly greater amount of protein (P: 5.5 g) and fat (F: 5.9 g) compared to cow (P: 3.4 g; F: 3.3 g), goat (P: 3.7 g; F: 3.8 g), and camel (P: 3.3 g; F: 4.0 g) milk [22,29]. Buffalo and reindeer milks also have a notably high lipid content (7.4 g/100 g and 16.1 g/100 g, respectively) [34]. In addition, mean lactose content varies modestly across ruminant milks at 4.51%, 4.75%, 4.79%, and 4.82% for 100 g of goat, sheep, buffalo, and cow milk, respectively [35]. See Figure 1 for a comparison of the differing macronutrient profiles of common animal milks, and Table 2 for a more detailed examination of the nutritional composition between different animal milks and plant-derived milk alternatives. It is noted that these cited values, and those in the following sections, should merely serve as typical examples of a milk’s nutritional composition. This caveat is raised with knowledge that milk composition can greatly vary under different conditions (i.e., protein composition is largely determined by genetics, and thus varies with herds; whilst lipid content is largely determined by environment, and thus varies with forage and season). Multiple studies with similar designs yet discordant findings have epitomised this variability of milk composition with animal breed, age, health status, diet, and lactation stage, or even milking yield/time of day [36,37]. For instance, the proportion of lipid content ascribed to either unsaturated or saturated FAs in mare (i.e., equine) milk can vary considerably across breed and lactation stage (unsaturated: 39–62%; saturated: 38–61%) [36]. Environmental pollutants may also alter the properties of milk fat [38]. Hence, great caution should be taken when drawing conclusions from a single study’s findings, especially when this is in the form of low-level evidence from analytical studies such as those cited above.

Besides gross protein, fat, and carbohydrate quantity, macronutrient quality can also greatly differ between milks of different origin, further altering implications for human health. Relative to monogastric mammals such as humans and mares, casein forms a far greater portion of the protein content in ruminant milk [22]. Moreover, comparisons across ruminant milks have documented that the casein fraction of sheep, goat, buffalo, and yak milk largely comprises β-casein, whilst α_S1_-casein predominates in cow milk [22,47]. Alongside α_S1_-casein and β-casein, α_S2_-casein and κ-casein complete the group of different casein phosphoproteins in mammalian milk. These four phosphoproteins are defined by their distinct primary amino acid (AA) sequence, micellar position, and subsequently, function (e.g., calcium and phosphate transportation, stability and solubility) [48]. Although whey, a by-product of the cheese-making process, is also a family of (five) heterogeneous and polymorphic protein fractions, the composition of whey is more consistent across species, with a common preponderance of β-lg, except in camel milk—in which serum albumin dominates, and β-lg is largely absent [22].

The unique classification of AAs and FAs composing individual milks has also sparked interest in the literature. Total and essential AA content are the highest in sheep and reindeer milk, whilst goat, buffalo, and yak milk still possess a higher composition than cow milk [22]. Regarding lipid profile, goat, sheep, and camel milk marginally surpass cow milk concentrations of monounsaturated (MUFA) and polyunsaturated (PUFA) FAs [49]. More notably, goat and sheep milk are considerable sources of short- and medium-chain triglycerides (MCTs), compared to long-chain triglyceride (LCT)-rich cow milk [50,51]. In exemplification of this, three MCTs (caproic acid, caprylic acid, and capric acid) even owe their names to the caprine species (i.e., goats). MCT content (C6:0–C12:0) (as a percentage of total fat content) is the highest in goat (23.0%) and sheep (21.8%) milk, followed by mare (15.2%), cow (10.5%) and human (7.3%) milk [37]. Potentially of further bioactive utility, the content of CLA (a PUFA found in animal milk) and vaccenic acid (a CLA precursor) is also higher in sheep milk (1.2% of total FAs) than cow (0.7%) and goat (0.6%) milk [52]. Finally, the oligosaccharide content of goat milk is approximately five and ten times greater than that of cow and sheep milk, respectively [53].

Beyond differences between mammalian milks, disparities between animal- and plant-sourced milks are more prominent still. The gross protein content of non-dairy milk beverages is typically only half that of cow milk [54]. Nonetheless, soy protein possesses a greater content of branched chain amino acids (BCAAs) than whey protein [55]. However, when using a reference evaluative means for assessing dietary protein quality in humans, the digestible indispensable amino acid score (DIAAS) [56] for milk protein is greater than that of soy protein [57]. Specifically, whilst cow milk protein possesses a DIAAS value ≥118% for all indispensable AAs, soy protein is limited by methionine and cysteine content with a lowest DIAAS of 90.6% despite having a DIAAS value >100% for most individual indispensable AAs [57,58]. Aside from protein content and quality, plant-based milk substitutes, except coconut milk, have lower levels of saturated fatty acids (SFAs) and greater levels of PUFAs and MUFAs than cow milk [54].

The literature has detailed the potential value of, inter alia, protein [59], particularly functional AAs [60]; MUFAs [61]; PUFAs [62]; MCTs [63]; and CLA [15] for improved cardiometabolic health. Hence, given the compositional differences outlined above, a robust assessment of the therapeutic potential of cow milk alternatives is required. The relationship between cow milk and glycaemia, blood pressure, and lipidaemia has been well-researched and reviewed in the literature [4], albeit with variable findings, yet investigations into how alternatively-derived milks influence acute postprandial and chronic fasting metabolism are limited. With a newfound importance emphasising the functional implications of individual foods and beverages for health, a review of non-bovine milk consumption is overdue.

### 3.2. Digestibility

Before progressing to any disparate effects of milk origin on cardiometabolic health, it is imperative to acknowledge that the protein and fat composition of different milks might also be variably digested. For example, the extent of gastric casein coagulation (or curd formation) alters the absorption of AAs, hence a higher ingested protein load may not necessarily translate to a higher delivered protein load. The acidification of α_S1_-casein in cow milk forms rigid and durable curds which are difficult to digest, whereas the coagula from goat, camel and mare milk are far more assimilable [22,64]. The degradation of β-lg also varies between species’ milk [22]. Goat [65] and sheep [66] β-lg is digested more efficiently than cow β-lg, but not as rapidly as mare β-lg [67]. Regarding lipid digestion, lipid globule size and FA chain length are two factors that may be inversely related to digestibility, thus influencing fat assimilation. Camel and goat milk are noted for small milk fat globules (MFG) compared to sheep, cow, and, most largely, buffalo milk with a mean diameter of 2.99 µm, 3.20 µm, 3.76 µm, 3.78 µm, and 8.7 µm, respectively [68]. However, the direct clinical consequences of MFG size for human health is still largely unknown, with a lack of long-term RCTs having been conducted [69]. Moreover, significant variation in mean MFG size with breed, herd, days in milk, season, and milking period has been reported [70,71]. Hence, this variability within each individual species’ milk devalues current comparisons of MFG size between different species’ milk. Finally, as the ester bonds of short- and medium-chain FAs are more readily hydrolysed than long-chain FAs, a greater fraction of the former, as found in goat and sheep milk, may contribute to higher digestibility [72].

## 4. The Impact of Milk Origin on Biomarkers of Cardiometabolic Health

### 4.1. Effects of Milk Origin on Energy Balance & Obesity

#### 4.1.1. Appetite Regulation

Both whole cow-milk product and the individual components of cow milk have long been evaluated for their efficacy in inducing satiety and modulating the release of satiety-related peptides (e.g., glucagon-like peptide-1; GLP-1) [73,74]. Indeed, there is some preliminary data from short-term RCTs in humans that have supported the conclusion that cow-milk proteins may promote greater satiety and subsequently induce a greater suppression of energy intake than alternative protein sources [75]. A single longer-term 23-week RCT has indicated that prolonged whey protein consumption may significantly reduce body weight and fat mass compared to an isoenergetic carbohydrate control, but not compared to other protein sources [76]; however, this has not yet been confirmed by other interventions.

More recently, acute clinical interventional studies have begun to assess the anorexigenic potential of cow milk alternatives. In an unblinded whole-milk product RCT, a direct comparison of how isovolumetric goat- and cow-milk-based breakfasts modulated subjective appetite, ghrelin release, and GLP-1 secretion was conducted [77]. Following consumption of the goat milk-based meals, participants reported a significantly decreased desire to eat and marked reduction in hunger, despite the energy content of the goat milk-based meal being 59 kJ lower than the cow milk-based meal. Moreover, a significant inverse correlation was observed between cumulative GLP-1 release and the area under the curve for both hunger and desire to eat after the goat milk-based breakfast only, whilst no significant differences were observed in ghrelin or GLP-1 levels at any single time point. However, it must critically be noted that the test meals in this study were composed of mixed dairy with both milk and cheese of either cow or goat origin being co-administered. Moreover, participants were not blinded in this open-label RCT. Contrastingly, a double-blind acute crossover study found no significant differences in GLP-1, cholecystokinin (CCK), ghrelin, or leptin secretion following the ingestion of either protein-fortified whole cow milk or protein-fortified whole goat milk [78]. Although this study found significant within-treatment suppression of prospective consumption at specific timepoints in both the goat and cow milk groups (relative to baseline), no significant difference in cumulative appetite response was detected between treatments. However, this current acute trial was not powered for the detection of differences in appetite measures with the primary outcome being postprandial plasma AA response (see ‘Aminoacidaemia’). Finally, the organoleptic aspects of (unfamiliar) milks must also be considered in clinical studies that aim to assess the impact of milk origin on appetite and subsequent energy intake. A downregulation of energy intake due to an aversion to a food’s pungent flavour or odour, as was speculatively observed following goat milk ingestion in a mouse study [79], should not inform the anorexigenic properties of a food.

A whole-milk product murine trial has shown that, after an initial energy restriction, leptin levels were significantly higher following renourishment with sheep or buffalo milk than with cow or goat milk [80]. This observed disparity in leptin secretion of mice may be explained by the varying lipid content of different milk types, or even by varying leucine content [81]. This latter notion corresponds with the above findings in mice [80], with leucine being present in greater amounts in sheep and buffalo milk than in cow and goat milk [35]. However, although there is some limited evidence of an inverse association between leptin concentration and subjective appetite during energy restriction in men [82], the wider research base does not support a causative role of circulating leptin in human appetite control. Moreover, clinical evidence surrounding the impact of milk consumption of different animal origin on leptin secretion is extremely limited.

Further consideration of how cow milk alternatives may influence appetite comes from lower-evidence-level research. For instance, an in vitro simulation study has investigated the satiety-inducing effects of whey samples from cow, goat, or sheep milk (or a 60:20:20 mixture of all three) as they are digested along the gastrointestinal tract [83]. Digested goat whey produced the highest secretion of GLP-1, whilst a fermented mixture of cow, goat, and sheep milk whey generated the greatest CCK response. Elsewhere, a hexapeptide has been located from β-lg that could potently inhibit dipeptidyl dipeptidase IV (DPP-IV) activity [84]. DPP-IV is an enzyme that catalyses the breakdown of incretin hormones, thus its inhibition results in a prolonged exposure of GLP-1 and glucose-dependent insulinotropic polypeptide (GIP), subsequently reducing glucagon release and increasing insulin secretion. Direct comparisons of the effectiveness of cow, goat, and sheep β-lg-derived peptides for the inhibition of DPP-IV have been conducted [85]. An in silico analysis indicated that sheep and goat whey could limit DPP-IV activity more efficiently than cow milk β-lg; however, this was not supported by a corresponding in vitro analysis. Sheep milk (6.5–8.5 g/L) contains a notably higher concentration of β-lg than buffalo (3.9 g/L), cow (3.2–3.3 g/L), and goat (1.5–5.0 g/L) milk [86].

Although the above studies provide some support to the acute anorexigenic utility of whey protein [87], it remains to be ascertained whether any clinically meaningful improvements in long-term appetite control can be gained from cow whey protein consumption, let alone from goat or sheep whey protein consumption. Moreover, it should also be remembered that the comparison of whey protein, which often only forms around 20% of the gross milk protein content, cannot solely determine the entire therapeutic potential of a given milk. A systematic review has summarised that there is some evidence that casein ingestion may actually induce a greater suppression of appetite in the long term [74].

#### 4.1.2. Energy Expenditure

Numerous studies of varying duration (i.e., short or long term) and strength of evidence (i.e., RCTs, epidemiological, animal, and in vitro) have investigated the effects of milk ingestion on appetite and energy intake. To a much lesser extent, researchers have addressed the possibility that milk consumption could enhance total and postprandial energy expenditure. To the authors’ current knowledge, there are no previous whole-milk product studies that have conducted intra-trial comparisons as to the influence of milk origin on metabolic rate. Nonetheless, this section will theorise on how the composition of different species’ milk could modulate postprandial energy expenditure.

Preclinical trials and clinical single-component randomised crossover studies alike have acknowledged that MCT consumption can potently elevate diet-induced thermogenesis, resting metabolic rate, and total energy expenditure relative to LCT consumption, under isoenergetic conditions [63,64,88,89,90]. An example of higher-quality evidence from an RCT neatly demonstrated a dose-dependent upregulation of daily energy expenditure by replacing varying amounts of LCTs with MCTs in their acute dose-response trial [91]. The observed increase in energy expenditure corresponded to a clinically meaningful mean change of +500 kJ/day with just small adjustments to the MCT:LCT ratio (15–30 g). Moreover, a longer-term 7-day overfeeding trial showed that postprandial thermogenesis was significantly higher among individuals who received an MCT-based liquid formula diet compared to those who received the LCT counterpart [92]. In this double-blind randomised crossover study, the thermic effect of food was 8.0% and 5.8% of total ingested energy for the MCT- and LCT-meals on day one, respectively. By day six, these values rose to 12.0% and 6.6%, respectively, demonstrating a greater thermogenic compensation with overconsumption of MCTs [92]. MCT assimilation putatively incurs a greater energy cost than LCTs due to elevated fat oxidation, reduced fat storage and heightened sympathetic nervous system (SNS) stimulation [63,64,91,93]. Consequently, substituting a milk with a higher MCT:LCT ratio for a less MCT-rich milk (e.g., goat milk for cow milk, 0.89 vs. 0.61 g total MCT/100 g milk, respectively) could enhance energy expenditure and thus aid weight maintenance [49,94,95] (see ‘Body Weight and Composition’).

Another single-component double-blinded RCT found that postprandial thermogenesis was significantly greater following whey protein (14.4%) than casein (12.0%) or soy (11.6%) protein ingestion [6], advocating the consumption of whey-dominant milk types (e.g., mare milk). Protein synthesis rate was two-fold higher in individuals following the ingestion of whey protein compared to casein protein [96], generating a greater direct thermogenic cost. However, indirect consequences of protein choice could also influence energy expenditure. For example, milks high in anabolic AAs such as leucine (e.g., sheep milk) could help to preserve or enhance lean body mass (LBM). Accordingly, LBM is closely related to fat-free mass, which is the single most important determinant of 24-h energy expenditure [97]. Hence, metabolic rate may be increased as a result [22,98,99].

#### 4.1.3. Nutrient Processing-Substrate Utilisation and Metabolic Efficiency

The role of individual milk components with regards to nutrient assimilation has been discussed in multiple review articles. Specific milk components that have been speculated to optimise nutrient delivery and metabolic efficiency include MCTs, CLA, carnitine, phosphorus, calcium, riboflavin, pantothenic acid, and milk sugars (lactose and galactose) [28,29,35,99,100]). However, for the most part, the credibility of these purported health claims remains to be confirmed through support from RCTs with gold-standard parameters of nutrient processing instituted as primary endpoints a priori. A critical outlook at the present level of evidence is overviewed here.

With a high content of C6:0, C8:0 and C10:0 (MCTs), murine trials have indicated that the partioning of goat milk minimises intestinal FA re-esterification, promoting the direct fueling of β-oxidation whilst attenuating protein oxidation [28,64,101] (see ‘Body Weight and Composition’). Yet, clinical superiority (or equivalence) trials are required to ascertain the metabolic fate of goat milk (fat), relative to cow milk (fat) in humans.

Calcium may also assist in lipid metabolism. Pertinently, cow milk is a salient source of calcium, yet sheep milk possesses a higher calcium concentration still. The most recent analysis reports calcium levels of 182, 130, 120, 93, and 28 mg/dL in sheep, goat, cow, mare, and human milk, respectively [37]. Buffalo milk is also an excellent source of dietary calcium [102]. Despite this, a pervasively low calcium intake persists across Asia, Africa, and South America [103]. Some, but not all, epidemiological studies have found significant inverse associations between calcium intake and body weight [104]. Moreover, a higher calcium intake has been linked with improved lipid metabolism in some preclinical [105] and clinical [106] RCTs. However, these early findings are contested by a more recent systematic review with a meta-analysis of RCTs which found that increased dairy-derived calcium intake does not influence body weight despite a possible facilitation of fat loss during shorter-term energy-restriction [107]. Thus, with the existing body of knowledge, it cannot currently be concluded whether an increased supply of calcium can optimise lipid mobilisation or minimise an individual’s risk of progressing towards a positive fat balance [99,107].

Lactose and galactose may also be linked with increased fat utilisation relative to other substrates. A 4-day single-component randomised crossover study conducted in a cohort of seven lactating and seven non-lactating women has indicated that the ingestion of a galactose beverage may stimulate the postprandial mobilisation and oxidation of endogenous fat whilst reducing protein oxidation, compared to an isoenergetic, isonitrogenous glucose beverage [108]. However, a more recent RCT among 12 (male and female) adults has found no differences in fat utilisation following a 60 g galactose load, compared to a matched glucose or fructose load [109]. Hence, the robustness of these findings needs to be assessed in a larger-scale clinical study. Moreover, an assessment of long-term trends regarding sugar-specific changes to lipid processing in humans is limited.

On the other hand, longer-term murine studies have found that the enrichment of diets with either of the above milk sugars instils a resilience to both fat and weight gain [110,111]. These findings may be explained by a galactose-induced stimulation of the SNS in epididymal fat depots, which would be expected to mirror an increased rate of energy expenditure in rats [110]. Although lactose content only varies marginally between the major ruminant milks (see Figure 1), levels of galactose, a key precursor of lactose, are more variable with concentrations of 4.0, 3.3, 0.6, and 0.3 mg/100 mL in cow, buffalo, goat, and sheep milk previously found, respectively [112]. However, whether cow milk ingestion yields a greater fat-oxidising capacity than non-bovine milk is yet to be assessed in clinical trials. Any future studies that do assess the impact of varying milk sugar content on substrate utilisation must also conduct a concurrent assessment of the postprandial blood glucose response to adjudge the glycaemic consequences of an elevated milk sugar content.

#### 4.1.4. Body Weight and Composition

Although the effects of cow milk consumption on body weight have been widely investigated in the literature [4], there is not as yet consensus on a positive relationship. In turn, far fewer studies have assessed the effectiveness of non-bovine milks for weight management. Nonetheless, some researchers have tracked changes in body weight following the administration of different species’ milk. An early RCT found that five months of goat milk ingestion resulted in significantly more weight gain compared to cow milk [113]. Whereas, another clinical RCT in Madagascan individuals reported no significant differences in weight gain between individuals receiving either cow or goat milk for 10 consecutive days [114]. However, it must be noted that the above two clinical trials were conducted in a cohort of undernourished children for whom weight gain was desirable, contrasting the status quo of many increasingly obesogenic societies.

A single-component randomised parallel RCT in 113 overweight individuals has shown that weight regain and fat accumulation following energy restriction was significantly lower with milk protein (calcium caseinate) supplementation, compared to an untreated control group [73]. However, this added protein was administered during a free-living weight maintenance phase during which time macronutrient intake was not measured and thus the energy content of the diets could not be precisely determined or matched. Nonetheless, this study reported enhanced weight control in the milk protein supplementation group, despite no estimated differences in energy intake.

Energy density has been reported as lowest in mare milk (1842–2051 kJ/kg), rising sharply with goat (3018 kJ/kg), camel (3283 kJ/kg), buffalo (3450 kJ/kg), and cow (3169–3730 kJ/kg) milk, before peaking in sheep milk (5932 kJ/kg) [70,115,116,117,118,119,120]. Hence, for any final benefit to weight management with the consumption of different species’ milk, these discrepancies in energy density would need to be fully offset by either a greater suppression of appetite and energy intake, or enhancement of energy expenditure. Therefore, an interesting question develops whether the increased protein content of sheep milk, for example, could sufficiently counterpoise its elevated energy content. However, it should also be considered that these cross-sectionally reported values of energy content are largely determined by fat content, which in turn is likely to vary with season (see ‘Composition’).

Preclinical whole milk product trials in murine models have echoed the above early clinical findings [113], showing greater weight gain following goat milk, as opposed to cow milk, intake [79,121]. Elsewhere, a disparate effect has been identified whereby 8-week camel milk administration resulted in significantly lower weight gain in non-diabetic rats, but significantly increased weight gain in streptozotocin (STZ)-induced diabetic rats, compared to a standard laboratory diet [122]. However, this study did not disclose the nutritional content of each feeding regime and it is therefore unknown whether the meals were isoenergetic.

To delineate which species’ milks merit further examination within the realm of weight management, some value can be gained by considering the underlying qualities of individual milks. As discussed in the preceding sections, milk components that might imply benefits for body and fat mass regulation include whey protein, casein protein, and AA content; MCT content; and mineral bioavailability [23,74].

Milk protein intake, generally, may optimise muscle mass, performance, and recovery, although these benefits are likely also contingent upon physical activity status [2,123,124]. Pertinently, the substitution of sheep milk for cow milk could optimise the proportion of daily energy intake ascribed to protein, in line with a commonly advocated high-protein antiobesity strategy [125,126,127,128]. Moreover, with greater concentrations of key AAs (e.g., leucine), sheep, buffalo, yak, and mare milk consumption could reinforce positive changes to body composition by conserving LBM during weight loss [35,98,99]. However, this theory remains to be substantiated with any empirical evidence from clinical studies. The administration of plant-based milk protein (e.g., soy protein) is presently unable to achieve the protein synthetic rate achieved by animal-based milks [129,130]. However, novel techniques such as unique plant breeding and anabolic AA fortification may soon reduce this imparity [131,132].

Caproic (C6:0), caprylic (C8:0), and capric (C10:0) acid undergo expedited hydrolysis before being transported directly to the portal circulation, whereas dietary LCTs typically undergo prolonged lipoprotein uptake and transport. Therefore, it is hypothesised that milk fat with a higher proportion of these MCTs (i.e., sheep and goat milk) may be more readily destined for β-oxidation rather than storage in adipose compartments whilst favouring protein synthesis and LBM retention [29,64,133,134]. However, this possibility is speculated in response to review articles, which themselves are largely based upon animal study findings.

Clinical trials providing higher-quality evidence are required to corroborate these claims. In an early review, the authors hypothesised that a preferential consumption of MCTs may be effective for weight management through an enhancement of either satiety or, more likely, postprandial thermogenesis, relative to LCT consumption [63]. However, in line with the findings of an acute RCT [135], a more recent review has concluded that great controversy still exists with regards to any effect of FA chain length on appetite [136]. Although these reviews place a greater emphasis on human studies, the focus largely remains on acute clinical trials, and prospective longitudinal RCTs are therefore needed to ascertain any potential antiobesity role of MCTs. Nonetheless, with multiple studies failing to show any effect of MCTs on appetite, relative to LCTs, any benefit of MCTs for weight management may more likely be derived through changes to energy expenditure (see ‘Energy Expenditure’). Finally, further trials would then be required to distinguish whether the consumption of individual milks with a higher content of MCT (i.e., sheep and goat milk) could translate to protection against obesity.

A plenitude of animal studies have reported that dietary CLA of ruminant origin, and primarily of the isomers cis-9, trans-11 and trans-10, cis–12, may enhance energy homeostasis and improve body composition [28]. Some evidence in mice alleges that the trans-10, cis-12 CLA isomer, specifically, possesses marked lipolytic properties, limits adipose formation and encourages the accretion of LBM [29,137,138].

In support of this, a significant inverse association was reported between plasma trans-10, cis-12 concentration and both body weight and serum leptin in an 8-week RCT among individuals with T2D [139]. However, this isomer is only present in modest amounts in dairy products compared to the predominant cis-9, trans-11 isomer [140], which was not found to be associated with body weight in the above 8-week RCT [139]. Moreover, a systematic review has reported that 0 out of 13 clinical intervention trials found a significant reduction in body weight, whilst only 3 out of 10 found a significant reduction in fat mass, consecutive to prolonged CLA consumption (>4 weeks) [141]. Thus, this poses doubt over therapeutic claims linking CLA of cow-milk origin with improved weight management, let alone the efficacy of milk-derived CLA from one animal origin versus another. Despite this, a later 6-month double-blind placebo-controlled RCT among 118 individuals with overweight or obesity, which utilised dual-energy x-ray absorptiometry, found site-specific reductions in fat mass with CLA consumption [142]. Moreover, in a more recent narrative review, it is postulated that CLA supplementation may have a beneficial impact on markers of body weight and/or adiposity [143]. However, with the current knowledge coming from trials of limited sample size and inconsistent study design, this verdict cannot yet be determined to be conclusive and remains largely controversial. Indeed, the need for longer-term RCTs evaluating the effect of CLA isomers on incidence of T2D and CVD is emphasised [5].

With a comparatively rich CLA composition (see ‘Composition’), it may be speculated that sheep-milk consumption could be preferable to that of cow milk for an improvement of body composition [142]. However, although CLA supplementation significantly reduced total body-fat mass in the above study [142], these changes were largely driven by regional-specific reductions in leg-fat mass. Moreover, it was controversially found that abdominal-fat mass was significantly reduced compared to baseline in the placebo group, but not among those receiving CLA supplementation. The deposition of fat in the lower limbs is likely a safer storage site compared to alternative, more central regions [144].

Finally, some, but not all, clinical studies have suggested that dairy-derived calcium may augment weight loss above that of supplemental calcium [99,145]. Insufficient calcium intake elicits a surge in plasma 1,25-dihydroxyvitamin D, which precedes an influx of calcium into adipocytes. This rise in intracellular calcium levels subsequently thwarts the breakdown of stored triglycerides by inhibiting the functioning of hormone-sensitive lipase [146]. However, as stated above in ‘Nutrient Processing’, a 2015 systematic review with meta-analysis has reported that an increased calcium intake, through either supplementation or a higher dairy intake, may not lead to a reduction in body weight in the longer term [107]. Nonetheless, with the knowledge that buffalo and sheep milk contain an especially high content of calcium (see ‘Nutrient Processing’), the roles of these milks could be an interesting area within future weight-management research.

## 5. Effects of Milk Origin on Insulinaemia, Glycaemia, and Type II Diabetes

### 5.1. Insulinaemia

It has long been suggested that acute diet-induced stimulation of insulin release may be beneficial for individuals unable to effectively maintain euglycaemia [147,148]. A randomised crossover study in nine healthy volunteers has assessed the insulinaemic response to whole cow-milk product [149]. These authors showed that ingesting 510 g of cow milk produced an insulin response not dissimilar to that of white wheat bread.

Besides lactose, the protein composition of milk has also been cited as an important determinant of postprandial insulin response following ingestion [74,77]. In addition to certain AA combinations, namely of arginine, leucine, and phenylalanine [150,151], a randomised crossover study among healthy participants has noted the insulinogenic effect of cow whey relative to cow casein and soy protein [6]. This finding has been supported in a narrative review [152] and in multiple RCTs among individuals with T2D [153,154]. Correspondingly, sheep milk contains a large amount of whey protein (around 10.6 g/L) and an unparalleled arginine, leucine, and phenylalanine content [22]. In addition, it has been reviewed that some, but not all, RCTs have found that whey protein more potently stimulates the release of both GLP-1 and GIP (key secretagogues of post-meal insulin), compared to alternative protein sources [74]. However, the studies in this review were all conducted in healthy, lean individuals.

There is also some lower-level evidence from in silico, in vitro, and animal studies which postulates that the capacity for peptides to potently inhibit DPP-IV may contribute to any antidiabetic potential of milk protein (see ‘Appetite Regulation’). This possibility has been recognised with the examination of camel- [155], cow- [84], and mare-derived [156] β-lg. In summary, the role that whey protein plays in the stimulation of insulin release among individuals with prediabetes and T2D following milk consumption is still largely unknown.

Milk is composed of a complex matrix of food components including low-GI carbohydrates; trans-palmitoleic FAs; and minerals, such as calcium, magnesium, and potassium. Multiple review papers have linked food components such as those described above with an amelioration of the postprandial insulin response [157]. Hence, with knowledge that the contents of such molecules vary with milk origin, it is expected that these discrepancies would impact the insulinotropic effect of a given species’ milk. For instance, palmitoleic acid content is greatest in mare milk (4.5%), followed by sheep (2.1%), goat (1.2%), and cow (1.0%) milk, whilst goat milk is the best source of potassium [37]. However, the alleged associations reported in such reviews are largely based upon data from either mechanistic animal studies using isolated food components or cross-sectional studies from which causality cannot be established. Thus, RCTs are required to determine the relationship between the molecules that make up the food matrix of milk and aspects of glucose control.

Although the insulinotropic effects of individual milk components have been scrutinised, few studies have explored the impact of whole milk product on insulin secretion. One randomised crossover study conducted among healthy Chinese men showed that the co-ingestion of soy milk with bread resulted in a significantly higher insulin response than the co-ingestion of cow milk with bread [158]. However, in a follow-up study by the same research group, it was revealed that cow milk may be equally as effective as soy milk for the regulation of blood glucose without the exaggerated insulin response, potentially owing to a greater GLP-1 response [159].

In terms of the effects of whole milk product from different animal origin on insulinaemia, knowledge from clinical experimental studies is extremely limited. An epidemiological study has suggested that rates of elevated fasting blood glucose, impaired glucose tolerance and T2D are significantly lower among the camel-milk-consuming communities of Rajasthan than the non-camel-milk-consuming communities [160]. Moreover, anecdotal reports in the literature have echoed these findings, observing the use of camel milk as an antidiabetic aid across Africa, Asia, and the Middle East [161,162]. However, a high risk of confounding is associated with these low-level-evidence observational studies. Stronger support comes from the findings of a 2-year RCT among individuals with type I diabetes (T1D) [163]. In this prospective study, participants who received 500 mL of whole camel-milk product daily in addition to their usual care experienced significant reductions in their insulin dose requirements compared to those receiving the usual care only. However, this RCT only recruited 12 participants into each group and neither the participants nor the researchers were blinded, making the study extremely vulnerable to demand characteristics. Thus, higher-quality evidence from large-scale RCTs must be attained before any robustness can be associated with these therapeutic claims for camel milk.

There is also some low-quality evidence from STZ-induced T1D murine models that camel milk consumption could improve glycaemic control [122]. A blunted gastric coagulation following the ingestion of camel milk protein has been postulated to increase the absorption of exogenous “insulin and/or ‘insulin-like’ proteins in camel milk” [122,161]. However, the sequencing of camel milk insulin has indicated that no differential effects are to be expected with regards to its susceptibility to proteolysis, compared to cow, goat, sheep, buffalo or human insulin [164].

Overall, there is a considerable paucity of studies which have conducted intra-trial comparisons regarding the influence of whole milk product from different origins on insulinaemia. However, a single acute randomised crossover study among 33 healthy participants was conducted which found no differences in postprandial insulin response or GLP-1 release following the administration of either a cow dairy- or goat dairy-based breakfast [77]. Longitudinal RCTs are needed to delineate how milk origin chronically influences insulin levels.

To the authors’ knowledge, the only within-trial assessment of how different species’ milk influences insulin levels alone comes from an animal study [80]. This refeeding study in energy-restricted mice showed that serum insulin levels were significantly increased following the administration of buffalo and sheep milk for one week, but not after one-week goat or cow milk administration. However, with this finding only being present in fasting insulin levels and knowledge of the notable physiological discrepancies between mice and humans, caution must be taken in the interpretation of these findings, as with any data cited from animal studies.

### 5.2. Glycaemia

As with postprandial insulinaemia (see ‘Insulinaemia’), to the authors’ knowledge, the only clinical RCT to have conducted direct comparisons of postprandial glycaemia following the consumption of whole milk product derived from different origins is the same study [77]. This acute unblinded crossover study assessed blood glucose for 3 h following the consumption of a mixed-meal of bread with milk and cheese from either cow or goat origin, however no significant differences were reported [77].

Despite a notable absence of clinical RCTs comparing whole-milk product from different animal origins, lower-quality evidence from human and animal studies assessing the glycaemic responses to individual milk components does exist. Although these studies lack the robustness to directly inform nutritional guidelines, they may provide some insight into research areas that are worthy of further investigation. These are discussed below.

Whereas whey protein possesses a superior insulinotropic capacity, findings from an acute randomised crossover study suggest that casein and soy protein can reduce postprandial blood glucose spikes without the exaggerated insulin response to whey [6]. This analogy promotes the consumption of ‘caseinic’ milks (i.e., ruminant milks) or even soy ‘milk’ for the regulation of postprandial hyperglycaemia. However, it should be noted that this clinical study recruited healthy, lean individuals and thus it is not known whether this response would be replicated among individuals with prediabetes and T2D.

In corroboration of the disparities that exist between the metabolism of rodents and humans, a murine trial recently demonstrated significantly improved glycaemic regulation following the administration of donkey (whey-dominant) milk compared to cow (casein-dominant) milk [165].

In terms of AAs, leucine has been pinpointed as a potent facilitator of glucose disposal [98]. Correspondingly, it has been reported that the leucine content in sheep, buffalo, yak and mare milk notably exceeds that of cattle, donkey, goat, and camel milk [35]. However, there are no clinical trials that have investigated the potential glycaemic consequences of this leucine variability using whole-milk product.

Some researchers have also speculated that CLA can stimulate the uptake and subsequent utilisation or storage of glucose, without inducing an excessive secretion of insulin [166]. However, these claims are not supported by the conclusions of recent systematic reviews that have collectively examined clinical RCT data [5,167]. Indeed, one double-blind RCT in men with obesity reported a pro-diabetic effect of the CLA isomer trans-10, cis-12 [168]. Yet, as noted previously, CLA found in milk is predominantly of the cis-9, trans-11 isoform and the utility of CLA for glycaemic regulation may be isomer-specific [169]. There are not currently any studies that have investigated any milk origin-specific effects of CLA, despite it being known that varying concentrations of CLA are present in goat, cow and sheep milk (see ‘Composition’).

Finally, and critically when discussing the glycaemic repercussions of any given food, milk is generally noted for its low GI [17,170]. GI is the two-hour incremental area under the curve (iAUC) for blood glucose following the ingestion of a food, relative to a standardised glucose (or white bread) load [171]. This desirable aspect of milk is primarily derived from its predominant major and minor carbohydrate portions of lactose and oligosaccharides, respectively (see ‘Composition’ for the lactose content of different species’ milk). Alternatively, plant-based milk substitutes, particularly coconut and rice beverages, which commonly reconstitute the absence of lactose with additional sugars or sweeteners, often have a considerably higher GI [172] (see Table 2). For instance, in a recent systematic review of international tables of GI values [17], reduced-fat cow milk was reported to have a mean GI value of 27 whilst GI values for coconut and rice milk beverages were as high as 68 and 92, respectively.

As a notable source of functional AAs, CLA and essential minerals, individuals unable to effectively maintain normoglycaemia could speculatively benefit from substituting in sheep milk for cow milk. Although, the glycaemic impact of an equicarbohydrate glucose load is attenuated when partially substituted for galactose, which is contrastingly found in greater quantities in cow milk, compared to sheep (and goat) milk [112,173].

### 5.3. Type II Diabetes

Despite a plethora of different trial designs (e.g., RCTs, observational, in vitro, and animal studies) documenting links between individual milk components (or occasionally whole milk product) and biomarkers of T2D, there remains a lack of high-quality evidence for a causal link between milk consumption and incidence of T2D [5]. A population-based epidemiological study, the CARDIA study, reported a dose-dependent inverse relationship between the inclusion of dairy products in the diet and the risk of overweight individuals developing insulin resistance syndrome over a 10-year period [1]. Moreover, a meta-analysis of 7 observational cohort studies including a total of 167,982 participants found that individuals with a high milk intake had a 13% reduced risk of T2D, compared to those with a low milk intake [174]. However, when these authors extended their analysis to examine sub-types of milk they reported that this benefit was specific to the fat content with the risk reduction being overturned with the consumption of full-fat milk [174]. This incongruity derived from the distinct compositions of different milk types highlights the critical importance of evaluating the effects of milk origin, and thus varying fat content (see Figure 1), on T2D risk. Accordingly, although the acute impact of milk origin on insulinaemic and glycaemic consequences has been somewhat explored (see ‘Insulinaemia’ and ‘Glycaemia’), to date, there are no prospectively-controlled longitudinal studies that have assessed the long-term antidiabetic utility of cow milk alternatives. Thus, in accordance with required future research into cow milk, it is reiterated that long-term human RCTs with T2D events as the primary endpoint are needed to determine whether milk consumption (of any origin) is causally related to T2D risk [5].

## 6. Effects of Milk Origin on Lipid Metabolism, Aminoacidaemia, and Cardiovascular Health

### 6.1. Lipidaemia

The relationship between milk lipids and markers of metabolic health is widely reported to be compound-specific [13]. Thus, with different lipid compounds present collectively in milk and in varying configurations across milks of different origin, it is important that the lipidaemic consequences of ingesting whole milk product are delineated.

A narrative review with meta-analysis has reported that short-term consumption of whole cow milk lipid may adversely increase LDL- and total cholesterol compared to substitution with either carbohydrates or unsaturated FAs [175]. However, these authors also reported that HDL-cholesterol may be increased and plasma triglycerides lowered—both protective effects. Consequently, this may result in an unaltered total:HDL-cholesterol ratio following cow milk intake (also proposed as a protective marker of coronary heart disease risk [176]). A more recent meta-review, although failing to account for possible confounding influence, reported no adverse impact of cow-derived SFAs on blood lipid markers when consumed as part of a food matrix such as milk [177]. Although this conclusion is in line with clinical observational studies, long-term RCTs are critically required before any causality can be established between cow milk consumption and CVD incidence [5].

As with postprandial insulinaemia and glycaemia (see respective sections above), only one trial has investigated the impact of whole milk (dairy) product from different species on postprandial lipidaemia [77]. This acute RCT found no significant difference in plasma triglycerides; total cholesterol; HDL-cholesterol; or non-esterified fatty acids (NEFA), which can indicate disrupted postprandial lipid metabolism; following the consumption of either a cow- or goat-dairy-based breakfast. From these findings it may be inferred that goat milk could prove to be a non-inferior substitute for cow milk in the regulation of postprandial lipidaemia. However, this particular study did not report LDL-cholesterol or total:HDL-cholesterol ratio and the overall impact of these meals (of cow or goat origin) on lipid profile can therefore not be established.

Although no further high-quality evidence is currently available from clinical studies (i.e., RCTs), a longer-term preclinical study found that incorporating goat milk into the diet of mice lowered levels of total and LDL-cholesterol, compared to cow milk ingestion [79]. Putatively, this benefit could be derived from an enriched MCT content in goat (and sheep) milk, compared to cow milk. As noted previously, MCTs may be hypocholesterolaemic relative to LCTs due to a rapid hydrolysis which bypasses adipogenesis [29,178].

Two recent studies using gas chromatography have also assessed the “lipid quality” of different species’ milk based upon an index of FA composition which they reported to determine the hypo- or hyper-cholesterolaemic nature of a milk [37,179]. However, whereas one study reported that goat and sheep milk were more cholesterologenic than cow and mare milk, the other reported the complete opposite [37,179] (see ‘Composition’ for the caveats surrounding compositional variability of milk).

In addition to lipid compounds, calcium, and the AAs carnitine and taurine have also been noted for their lipid-lowering capability [22,28,70]. However, as with the evidence cited in these reviews, this purported relationship is largely based upon either observational data or findings from animal studies which have not exclusively used the dairy-derived forms of these compounds. Hence, these reports are far from conclusive. Regardless, it is worthy to note the content of these compounds across different species’ milk. Similarly to calcium (see ‘Nutritional Processing’), total carnitine content is the highest in sheep milk (943 µmol/L), followed by cow (169 µmol/L), goat (136 µmol/L), mare (75 µmol/L), and human (65 µmol/L) milk [180]. Sheep milk also possesses taurine in concentrations (140 µmol/L) closest to that of human milk (300 µmol/L), with mare (30 µmol/L) and cow (10 µmol/L) milk proving inferior sources [22,70,181]. Hence, the above in vitro analyses could indicate a desirable composition of sheep milk for the postprandial absorption and disposal of lipids towards oxidative pathways, however longer-term RCTs must first be conducted to confirm any benefits from the consumption of these milk-derived compounds.

### 6.2. Aminoacidaemia

The digestion and absorption of AAs in milk has both direct and indirect effects on the regulation of metabolic health through the stimulation of insulin release, upregulation of rate of protein synthesis, and, to a lesser-known extent, the suppression of postprandial appetite.

The prolonged release of nutrients following casein ingestion, as opposed to the rapid breakdown of whey protein, is reflected in the dynamics of aminoacidaemia. Acute postprandial RCTs have shown that casein induces a sustained appearance of plasma AAs, whilst whey protein triggers a higher yet transient spike in plasma AA concentration [96,182]. The elongated aminoacidaemic response of casein is reported to manifest in a modest augmentation of protein synthesis (+31%) despite reduced leucine oxidation and stunted whole-body protein breakdown [96]. Conversely, the sharp rise in plasma AAs following whey consumption resulted in a superior muscle protein synthesis (+68%) despite an uninhibited whole-body protein breakdown and upregulated leucine oxidation [96]. However, more recent studies have demonstrated that although whey protein ingestion produces greater peaks in plasma AA concentration, this may not necessarily translate to a higher rate of myofibrillar protein synthesis [182,183].

In addition to whey and casein, free amino acid (FAA) concentrations also differ greatly between species’ milk, more notably than total AA composition [22]. For example, FAAs, which possess superior absorbability to their protein-bound counterparts [184], are found in greater concentrations in mare milk (1960 µmol/L) compared to cow milk (578 µmol/L) [181]. Elsewhere, higher levels of FAA have been reported at 3913, 1999, 1357, and 1061 µmol/L in mare, goat, sheep, and cow milk, respectively [185]. Additionally, it has been argued that AAs from goat milk may be more readily available for utilisation than cow-milk protein, due to goat curds being softer and more friable when acidified [186]. However, there remains to be direct experimental evidence in humans to support these claims.

A whole-milk product randomised crossover study has found similar patterns of total, branched-chain, and essential plasma AA appearance following the ingestion of fortified milk drinks from either cow or goat origin [78]. However, the iAUC for valine and citrulline were significantly higher following goat milk ingestion, whereas the iAUC for tyrosine, and the leucine and isoleucine concentrations at given timepoints, were significantly higher following the cow-milk beverage. Subsequently, these researchers also conducted a whole-milk product double-blind RCT to assess plasma AA appearance following sheep- and cow-milk ingestion [187]. This trial was the first study of its kind to do so in humans and highlights the rising interest in the utility of non-bovine milks for the management of metabolic health. Pertinently, sheep milk consumption resulted in a significantly greater concentration of certain AAs compared to cow milk, notably all three proteinogenic BCAAs, but also lysine, methionine, and proline [187]. However, it should be noted that the participants in this study were all habitual avoiders of milk, with 80% self-identifying as lactose intolerant. Hence, it is not known whether the same results would be found among individuals who habitually drink (cow) milk. Further clinical RCTs are required, not only to determine the effects of milk origin on aminoacidaemia among lactose-tolerant individuals, but also to assess how any changes in AA response might impact protein fractional synthetic rate.

In summary, milks of varying FAA concentration, gross casein:whey ratio and casein or whey sub-components are anticipated to engender distinct aminoacidaemic consequences. However, further clinical trials are required to determine whether these putative changes translate to an improvement in acute and chronic biomarkers of metabolic health.

### 6.3. Cardiovascular Health

Largely owing to the SFA content of dairy products, the relationship between milk intake and CVD has long sparked contention in the literature. Although early research suggested that undesirable long-term cardiovascular consequences may arise from continued milk-fat consumption, this relationship is far more complex than previously envisioned [175,188,189]. Eclipsing any LDL-elevating effect of SFAs, complex milk composition far exceeds the impact of a single nutrient and implications for CVD should be considered for milk as a whole [5,37]. Pertinently, the majority of observational studies now actually oppose prior suggestions that dairy intake is positively and adversely associated with CVD risk. Indeed, a recent review appraising the current body of epidemiological evidence concluded that whole-fat dairy does not increase risk of CVD [190]. Similarly, in another review with a greater focus on liquid milk, the authors collated findings, largely from observational studies, demonstrating a negative association between whole-milk product and CVD risk, especially for stroke and hypertension [4]. However, corresponding data from RCTs is far more limited and although some studies have reported no adverse effects of milk consumption on CVD risk, these findings are predominantly either from acute intervention studies or from studies employing surrogate markers of CVD [5]. Hence, there is a persisting need for gold-standard evidence in the form of long-term RCTs with cardiovascular events as the primary endpoint in order to confirm any causal relationships between milk intake and CVD incidence.

More pertinently, there are currently no longitudinal observational studies or RCTs that have compared the long-term cardiovascular consequences of consuming whole milk product from one origin versus another. Thus, no consensus can currently be realised to form the basis of nutritional recommendations regarding the intake of different species’ milks for better cardiovascular health. Nonetheless, the following section collates the existing key findings from studies of ranging evidence quality and discusses how the varying compositions of milk from different origins could theoretically influence an individual’s risk of developing CVD.

The differences that are evident in the composition of different species’ milks (see ‘Composition’) could prove invaluable for the amelioration of cardiovascular health. Some researchers have produced lipid quality indices to detail and compare the potential influence of milk origin on lipoprotein metabolism (see ‘Lipidaemia’). These have been used to estimate the atherosclerotic and thrombotic risk of consuming milks of different origin. Accordingly, utilising gas chromatography, a 2019 analytical study performed comparisons between cow-, sheep-, and goat-milk samples [179]. With a notable source of PUFAs and n-3 FAs, sheep milk had the lowest index of thrombogenicity, whilst, with a high short-chain fatty acid (SCFA) and minimal SFA content, goat milk produced the lowest index of atherogenicity. However, one year later, the above lipid quality indices were reported to be optimal in mare milk, bettering the alternative species’ milks in the contrasting descending order of cow, goat, and sheep milk [37]. With both of these studies being conducted in Poland, this discrepancy emphasises the variability in milk composition with seasonal variance, breed diversity, lactation phase, etc. (see ‘Composition’). With knowledge that goat and sheep milk desirably contain higher concentrations of MUFAs, PUFAs, and MCTs, the 2019 findings [179] may possess greater external validity; however, this remains to be tested. With a lipid profile rich in linoleic (C18:2, n-6) and α-linolenic (C18:3, n-3) acids and a minimal palmitic (C16:0) and stearic (C18:0) acid content [191,192], the utility of mare milk should also be assessed for individuals with an increased risk of CVD.

The adverse association between high blood pressure and poor cardiovascular health in humans has been well-recognised in longitudinal observational studies and RCTs alike [193]. Pertinently, there is also an accumulating body of evidence from moderate-term RCTs [194,195] and review articles [132,196] that cow-milk peptides could have the potential to improve blood pressure, possibly, although evidence is lacking in clinical trials, through the inhibition of angiotensin-converting enzyme I (ACE). However, the majority of this work has been conducted among overweight individuals and it is therefore currently difficult to untangle any direct antihypertensive effect of milk peptides from the possible indirect benefit gained from body weight/fat loss [5].

Although evidence from RCTs comparing the antihypertensive utility of cow-milk alternatives is absent, some data from compositional analyses have acknowledged sheep and goat milk as a potential source of ACE-inhibitory peptides [120,197]. Another compositional study directly comparing the ACE-inhibitory capacity of sheep and cow milk products revealed that sheep milk peptides inhibited ACE activity by 50% (IC50) at a lower concentration [198]. Although the authors could not explain this heightened potency, it was speculated to be associated with an elevated proteolysis of sheep milk peptides compared to cow milk peptides.

Also, β-lg-derived peptides have generated antihypertensive promise in animal and in vitro trials [199], theoretically supporting a role for sheep-milk consumption (see ‘Appetite Regulation’); however, robust clinical evidence is limited. Assay techniques have also revealed that ACE-inhibitory peptides are produced from the proteolysis of β-casein, which again is found in higher quantities in sheep, goat, and buffalo milk than cow milk [22,28,200,201].

Finally, incorporating potassium, magnesium, and calcium into the diet whilst restricting sodium intake has been reviewed to ameliorate blood pressure control in some, but not all, clinical studies [202,203]. However, RCTs are required to assess whether these compositional differences can translate to meaningful disparities in biomarkers and incidence of CVD following milk consumption of different origin.

## 7. Conclusions

The effect of milk origin on cardiometabolic health is an emerging area of research. There is some data, although primarily from compositional analyses [35,37], in vitro studies [83], animal studies [80], and acute clinical RCTs [77,78,187], that milk from non-bovine origin (notably sheep and goat milk) could prove to be a viable substitute to cow milk for the maintenance, or even enhancement, of cardiometabolic health. However, a collation of the compositional differences and postulated therapeutic utility, as presented in this review, indicate that the level of evidence required to form nutritional recommendations surrounding milk origin is currently lacking. Nonetheless, there are some interesting results, albeit largely from preliminary studies, that have generated excitement around sheep milk consumption for the possible attenuation of cardiometabolic risk. This interest is largely based upon its favourable profile of lipids (e.g., MCTs, CLA), protein (e.g., leucine), and minerals (e.g., calcium). In theory, these compounds could provide protection from obesity, T2D, and CVD through the modulation of postprandial glycaemia, lipidaemia and aminoacidaemia; nutrient processing; postprandial thermogenesis; and/or appetite. Comparably, with desirable nutritional compositions and some promising early findings, goat and buffalo milk may also prove to be robust alternatives to cow milk. However, as with sheep milk, there is currently a stark absence of high-quality research in humans. Hence, as remains pertinent for cow milk, to substantiate any claims that the consumption of cow-milk alternatives can improve cardiometabolic health, causal data from long-term clinical RCTs, ideally with T2D and/or CVD events as the primary endpoint, are required. Evidence from large-scale studies that support the conjectures formed in this review could not only be of value to individuals allergic or intolerant to cow milk, but potentially also to those at an increased risk of cardiometabolic disease. Thus, this review concludes that further exploration into the therapeutic potential of milk beyond the realms of cow dairy is warranted.

## Figures and Tables

**Table 1:** Mean contribution of individual species’ milks towards global production [24].

Milk Origin	Global Milk Production (%)	Global Milk Production (kg)
Cow	81.3	714,400,000,000
Buffalo	14.8	130,300,000,000
Goat	2.2	18,900,000,000
Sheep	1.3	11,800,000,000
Camel	0.4	3,200,000,000

Values rounded to nearest 0.1 percent or 10^9^ kg.

**Table 2:** Nutritional composition per 100 mL milk of different animal-derived milk and plant-based milk alternatives.

	Milk Origin									
	Cow	Buffalo	Sheep	Goat	Equine	Camel	Soy	Oat	Rice	Almond
Total fat (%)	3.3	7.4	5.9	3.8	1.1	4.0	2.0	2.2	1.0	1.1
MCT (% of total fat)	10.5	7.1	21.8	23.0	15.2	1.5	n.d.	n.d.	n.d.	0.2
CLA (% of total fat)	0.7	0.5	1.2	0.6	0.1	0.9	n/a	n/a	n/a	n/a
SFA (% of total fat)	68.4	70.8	65.0–75.0	65.0–73.8	38.0–61.0	66.1	14.3	18.9	12.0	22.6
MFG diameter (µm)	3.8	8.7	3.8	3.2	2.8	3.0	n/a	n/a	n/a	n/a
Total protein (%)	3.4	4.4	5.5	3.7	1.8	3.3	2.6	1.0	0.5	0.6
Casein:whey	82:18	82:18	76:24	78:22	52:48	73:27–76:24	n/a	n/a	n/a	n/a
Lactose (%)	4.8	4.8	4.8	4.5	6.9	4.3	n/a	n/a	n/a	n/a
Galactose (%)	4.0	3.3	0.3	0.6	<0.1	<0.1	n/a	n/a	n/a	n/a
GI (0–100)	27–37	-	-	-	89.3 (donkey)	-	31–37	69	79–92	49–64
Energy (kJ)	316.9–373.0	345.0	593.2	301.8	184.2–205.1	328.3	179.9	195.8	225.9	126.8
Calcium (mg)	119.8	183.9	181.7	130.4	92.9	106.0	113.0	120.0	118.0	160.0
Potassium (mg)	145.0	101.6	120.0	181.0	50.5	156.0	122.0	162.0	27.0	67.0

Data obtained and collated from a range of supermarket product labels and/or the following sources in the literature [17,22,28,29,34,35,39,40,41,42,43,44,45,46]. Equine milk refers to mare milk unless otherwise specified. n.d., not detected.

## Data Availability

Not applicable—no original data were reported in this review.
